# Chemical composition and some biological activities of the essential oils from basil *Ocimum* different cultivars

**DOI:** 10.1186/s12906-017-1587-5

**Published:** 2017-01-19

**Authors:** Arpi Avetisyan, Anahit Markosian, Margarit Petrosyan, Naira Sahakyan, Anush Babayan, Samvel Aloyan, Armen Trchounian

**Affiliations:** 1 Nairian CJSC, Khorenatsi 15, Yerevan, Armenia; 2 Department of Biochemistry, Microbiology & Biotechnology, Biology Faculty, Yerevan State University, 1 A. Manoogian Str., 0025 Yerevan, Armenia

**Keywords:** *Ocimum*, Essential oil, Methyl-chavicol, Linalool, Nerol, Citral, Antioxidant, Antibacterial activity

## Abstract

**Background:**

The plants belonging to the *Ocimum* genus of the Lamiaceae family are considered to be a rich source of essential oils which have expressed biological activity and use in different area of human activity. There is a great variety of chemotypes within the same basil species. Essential oils from three different cultivars of basil, *O. basilicum var. purpureum*, *O. basilicum var. thyrsiflora*, and *O. citriodorum* Vis*.* were the subjects of our investigations.

**Methods:**

The oils were obtained by steam distillation in a Clevenger-type apparatus. The gas chromatography mass selective analysis was used to determine their chemical composition. The antioxidant activities of these essential oils were measured using 1,1-diphenyl-2-picrylhydrazyl assays; the tyrosinase inhibition abilities of the given group of oils were also assessed spectophotometrically, and the antimicrobial activity of the essential oils was determined by the agar diffusion method, minimal inhibitory concentrations were expressed.

**Results:**

According to the results, the qualitative and quantitative composition of essential oils was quite different: *O. basilicum var. purpureum* essential oil contained 57.3% methyl-chavicol (estragol); *O. basilicum var. thyrsiflora* oil had 68.0% linalool. The main constituents of *O. citriodorum* oil were nerol (23.0%) and citral (20.7%). The highest antioxidant activity was demonstrated by *O. basilicum var. thyrsiflora* essential oil. This oil has also exhibited the highest tyrosinase inhibition level, whereas the oil from *O. citriodorum* cultivar demonstrated the highest antimicrobial activity.

**Conclusions:**

The results obtained indicate that these essential oils have antioxidant, antibacterial and antifungal activity and can be used as natural antioxidant and antimicrobial agents in medicine, food industry and cosmetics.

## Background

The plants belonging to the basil genome or *Ocimum* genus of the Lamiaceae family are aromatic ones [1] and are considered to be a rich source of essential oils-the metabolites, synthesized by plants for specific functions, using various secondary metabolic pathways. Humans have learned to use these metabolites since antiquity for food preservation, flavoring, and as medicine. The basil essential oils are usually extracted from the leaves and flowering tops of basil plants. Through the centuries basil was cultivated for culinary and medicinal purposes in many countries, which created a great diversity of species within the *Ocimum* genus: the genus *Ocimum* comprises more than 150 species and is considered as one of the largest genera of the Lamiaceae family.

It is known, that different cultivars of basil have the genetic ability to generate and keep different sets of chemical compounds. This ability leads to a great variety of chemotypes within the same basil species. According to some investigations [2], the essential oils distilled from various basil cultivars can contain alcohols (linalool), oxides (1,8-cineole), phenols (eugenol, methyl eugenol, methyl isoeugenol, thymol), esters (methyl cinnamate), aldehydes (citral), and camphor. The 1,8-cineole, methyl cinnamate, methyl chavicol, and linalool are constituents responsible for the distinct aroma of basil plants [3].

Lawrence [4] named four major chemotypes of basil: methyl chavicol-rich, linalool-rich, methyl eugenol-rich, and methyl cinnamate-rich. Both methyl chavicol and methyl eugenol are phenylpropanoids produced by shikimic acid pathway and are reported to be toxic to insects and microbes. Linalool is a terpenoid produced by mevalonic acid pathway and known to possess antioxidant and antimicrobial activity [5]. Methyl cinnamate is the methyl ester of cinnamic acid. It is found naturally in many aromatic plants, including fruits like strawberry and is known to attract pollinators. According to Marotti et al. [6] the European basils are mostly of linalool and methyl chavicol types, whereas tropical basils have methyl cinnamate as their major constituent. Basils of methyl eugenol chemotype could be found growing in North Africa, Eastern Europe, and parts of Asia [7].

Numerous papers have been published on the antimicrobial and antioxidant properties of basil essential oils and its constituents. Koeduka et al. [8] and Zabka et al. [9] reported the antimicrobial activity of eugenol with analgesic properties for humans. Liu et al. [5] investigated the antioxidant and antimicrobial activity of linalool and geraniol. While Soković et al. [10] and Huang et al. [11] investigated the usage of linalool, methyl chavicol, and thymol for skin protection against all sources of environmental skin aggressors and treatment of various dermatological disorders.

Since the chemical composition (chemotype) and biological activity of essential oils distilled from the plants belonging to the same species may vary significantly, depending on the variety of cultivars, environment, elevation and cultivation methods, it is interesting to study the essential oils obtained from the different kinds of basil grown in Armenia, in similar conditions, at a significant elevation (1600 m above sea level).

In the present study the comparative analysis of the chemical composition and biological activities of essential oils distilled from three varieties of basil, *O. basilicum* var. *purpureum*, *O. basilicum* var. *thyrsiflora*, and *O.* x *citriodorum,* was carried out*.* The plants under investigation were grown in the same soil, at the same elevation, and under the same climatic conditions. The first two cultivars were varieties of *O. basilicum* specie*s*, or Sweet basil, and the third one, the Lemon basil (*O. x citriodorum*) was a hybrid between *O. basilicum and O. americanum*. The purpose of this paper was also to study the biological activities of given oils and to evaluate their potential using in food industry, cosmetics and medicine.

## Methods

### Plant material

The three basil cultivars (*O. basilicum* var. *purpureum*, *O. basilicum* var. *thyrsiflor*a, and *O.* x *citriodorum*) were grown from the seeds sown in the greenhouse, with subsequent transplantation of the seedlings to the same field, in the Kotayk Region of Armenia, where they have been growing side by side, at an elevation of 1600 m above the sea level. Plant materials were collected during blossoming period (July–August, 2014). The plant materials were identified at the Institute of Botany, National Academy of Sciences of Armenia, Yerevan (Armenia). The plants were not included in the herbarium as there were cultivated species and not typical for the flora of Armenia. The samples of basil cultivars are available at the Department of Microbiology & Plants and Microbes Biotechnology, Biology Faculty, Yerevan State University, Yerevan, Armenia.

### Essential oil extraction

Essential oils were extracted from air dried plant material (aerial parts only) by hydro-distillation, using a Clevenger-type apparatus and lasted 3 h. The distilled essential oils had been dehydrated with anhydrous sodium sulphate and stored at 4 °C in dark airtight bottles until further analysis [12].

### Determination of essential oil chemical composition

The gas chromatography (GC) mass selective (MS) analysis of the essential oils was performed using a Hewlett–Packard 5890 Series II gas chromatograph, fitted with a fused silica HP – 5MS capillary column (30 m × 0.25 mm, in thickness 0.25 μm). The oven temperature varied from 40–250 °C with the scanning rate of 3 °C/min. Helium (purity 5.6) was used as a carrier gas at a flow rate of 1 mL/min. The GC was equipped with Hewlett–Packard 5972 Series MS detector. The MS operating parameters were ionization voltage 70 eV and ion source temperature 250 °C. The diluted samples of essential oils (1/100, v/v in HPLC methanol) of 1 μL had been injected manually. To avoid overloading the GC column, the essential oils were diluted 1:100 (v/v) in methanol. The identification of peaks was tentatively carried out based on library search using National Institute of Standards and Technology (NIST)-2013. Relative Retention Index (RRI) was calculated for HP-5MS column. For RRI calculation a mixture of homologues *n*-alkanes (C9-C18) was used under the same chromatographic conditions as for analysis of the essential oils.

### Investigation of antimicrobial activity by agar diffusion method

The antibacterial and antifungal activity of the essential oils was determined by the agar diffusion method [13]. This method was preferred over the dilution method because of low solubility of essential oils in water and in meat peptone broth. The following concentrations of essential oils were used: 150; 100; 50; 25; 12.5; 6.25 μL/mL; dimethyl sulfoxide (DMSO) was used as the solvent. The 100 μL of each oil solution was introduced to the wells in the agar with test microorganisms. Different Gram-positive (*Bacillus subtilis* WT-A, isolated from metal polluted soils of Kajaran, Armenia; *Staphylococcus aureus* MDC 5233 (Microbial Depository Center, Armbiotechnology Scientific and Production Center, Armenia; laboratory control strain) and Gram-negative (*E. coli* VKPM-M17 (Russian National Collection of Industrial Microorganisms at the Institute of Genetics and Selection of Industrial Microorganisms, Russia; laboratory control strain), *Pseudomonas aeruginosa* GRP3 (Soil and Water Research Institute, Iran) bacteria and ampicillin-resistant *E. coli* dhpα-pUC18 were used. Bacterial cultures were grown on Mueller-Hinton agar. Ampicillin (25 μg/mL) as a positive control and DMSO as a negative control were used. The yeasts (*Candida albicans* WT-174 isolated from infected vaginal microbiota of hospitalized patients (clinical strain) and *Debariomyces hansenii* WT (French National Institute for Agricultural Research, France; laboratory control strain) were grown and maintained on Sabouraud-dextrose agar for 24 h at room temperature. As the positive control fluconazole (25 μg/mL) was used. Data were expressed in minimal inhibitory concentrations (MIC) values.

The selected pieces of nutrient medium from the zones of microorganism growth absence were transferred to the nutrient medium corresponding to each microorganism and then they were incubated for 2–3 days at appropriate temperature to determine the bacteriostatic or bactericidal action of the oils. The action of oils is evaluated as bacteriostatic in case of renewed growth of test-microorganisms after the re-cultivation.

### Determination of radical scavenging activity

Free radical scavenging ability of the essential oils was tested using ethanol solution of 1,1-diphenyl-2-picrylhydrazyl (DPPH) [14]. Catechin was used as a positive reference. Sample solution contained 125 μL (1 mM) DPPH, 375 μL ethanol and 500 μL of test-solution (essential oils or catechin with different concentrations). In the control solution the test-solution was replaced by ethanol. The absorbance was measured at the wavelength of 514 nm.

The radical scavenging activity was calculated using the following formula: Radical scavenging activity (%) = Ac–As/Ac × 100, where Ac is absorbance of control (DPPH without the addition of test solution), and As-the absorbance of the sample.

IC_50_ calculated denote the concentration of investigated samples required to decrease the DPPH absorbance at 514 nm by 50%.

### Tyrosinase inhibition colorimetric assay

Tyrosinase inhibition colorimetric assay was carried out according to the method, as described [15, 16]. Each essential oil was dissolved in DMSO to obtain concentration of 20 mg/mL. These stock solutions were diluted to 600 μg/mL concentration in 50 mM potassium phosphate buffer (pH 6.5). Arbutin acid was prepared in similar way and used as positive control. 700 μL of each sample solution or positive control were combined with 300 μL of mushroom tyrosinase (333 Unit per mL in phosphate buffer, pH 6.5). After incubation at 20–22 °C for 5 min, 1100 μL tyrosine (2 mM) were added to each well. Plates were incubated at room temperature for 30 min and the absorbance was measured at the wavelength of 492 nm using the spectrophotometer Genesys 10S UV–vis (Thermo Scientific, USA). Percent inhibition of tyrosinase activity was calculated according to the formula: inhibition (%) = 100-(W_sample_/W_blank_) × 100, where W is absorbance at 492 nm. W_blank_ is absorbance of control reaction (containing all reagents without test compound).

### Statistical analysis

Experimental data (*n* = 4) were expressed as means with standard errors. The latter did not exceed 3% (if not indicated). The validity of differences between experimental and appropriate control data were evaluated by Student’s criteria (P) using Microsoft Excel 2010 with the help of *T* test function; *P* < 0.05 (if not indicated).

## Results

### Determination of chemical composition of essential oils

The results from the quantitative and qualitative analysis of essential oils constituents are presented in Table 1: the average yield of the essential oils was 0.2%. More than 40 compounds were isolated, detected and most of them identified for each essential oil sample. The dominant components were identified to be linalool, methyl chavicol, citral and nerol.

**Table 1:** Chemical composition of essential oils of *Ocimum basilicum* var*.purpureum*, *Ocium basilicum* var*. thyrsiflora*, *Ocimum citriodorum*

Chemical components	Relative Retention Index^a^	*O. basilicum* var. *purpureum,* %^b^	*O. basilicum* var. *thyrsiflora,* %	*O*. x *citriodorum,* %
1-octen-3-ol	979	0,2	-	0,1
1-8- Cineole	1035	1.40	3.50	-
(Z) -β-Ocimene	1058	-	-	0.24
γ-Terpinene	1078	-	-	0.22
Fenhone	1089	-	-	0.32
Linalool	1100	18.00	68.00	9.42
Camphor	1146	1.30	1.35	-
α - Terpineol	1181	-	-	0.62
Methyl chavicol	1203	57.3	20.00	9.45
Nerol	1231	-	-	23.00
Neral	1244	-	-	4.93
Geraniol	1259			5.20
Geranial	1274	-	-	15.77
Bornyl acetate	1291	0.13	-	-
Neryl acetate	1321	-	-	0.65
Methyl cinnamate	1338	-	-	0.49
β-Elemene	1387	3.62	0.67	0.53
β -Caryophyllene	1419	1.72	-	7.80
β –Copaene	1428	0.28	-	0.56
trans-α-Bergamotene	1433	4.34	1.34	3.52
α-Humulene	1455	0.55	0.28	1.52
cis- β-Farnesene	1472	0.31	-	0.48
Germacrene d	1482	0.68	0.17	-
β-Cubebene	1497	-	0.75	2.26
α-Bulnesene	1502	1.39	0.68	0.47
α-Amorphen	1510	1.54	0.69	-
δ-Cadinene	1518	-	-	0.38
Aromadendrene	1529	1.67	0.28	-
Spathulenol	1544	0.68	-	-
Caryophyllene oxide	1550	0.57	-	-
α –Bisabolene	1561	-	-	2.29
β - Bisabolenene	1572	-	-	8.31
α-Bisabolol	1642	-	-	0.29

^a^for HP-5 capillary column

^b^%: Calculated from MIC data

According to the data obtained, *O. basilicum* var. *purpureum* contains 57.3% methyl chavicol, with the second largest component being linalool (18%). This places the given variety of *O. basilicum* into methyl chavicol-rich chemotype. *O. basilicum* var. *thyrsiflora* belongs to linalool-rich chemotype, with concentrations of linalool and methyl chavicol being 68 and 20% respectively. These data are in a good accordance with the results reported by Sishu et al. [17]. For the essential oil from *O.* x *citriodorum* species the predominant constituents were identified to be citral (21%) and nerol (23%), therefore it could not be classified as belonging to any of the chemotypes mentioned above, but will rather form its own, nerol-rich chemotype. The data on *O.* x *citriodorum* are somewhat consistent with the similar results published by Carović-Stanko et al. [18] on essential oil distilled from the plant of the same species, except for the fact that there were more than 45 constituents of *O.* x *citriodorum* essential oil identified in the present study, as opposed to 20 components identified by Carović-Stanko et al. [18].

### Antimicrobial activity of essential oils

**Fig. 1:**
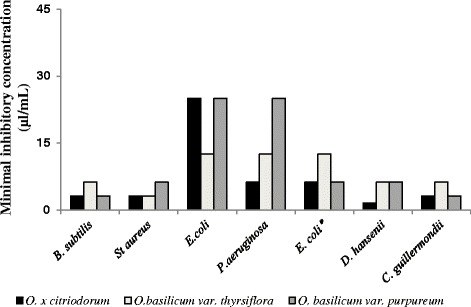
The minimal inhibitory concentrations (MICs) of *O. citriodorum*, *O. basilicum* var. *thyrsiflora* and *O. basilicum* var. *purpureum* essential oils on selected Gram-positive, Gram-negative bacteria and fungi. Antibiotic-resistant *E. coli* dhpα-pUC18 strain was used. For bacteria and fungi strains and other details, see Methods. *Antibiotic-resistant *E. coli* dhpα-pUC18 strain

The present investigation revealed that Gram-positive bacteria tested were more sensitive to all three essential oils than Gram-negative bacteria (Fig. 1). Such tendency is also observed by other authors [19]. The essential oil of *O.* x *citriodorum* was quite active against *B. subtilis* and *St. aureus, with the* MIC of 3.125 μL/mL. The same MIC was recorded for the essential oil of *O. basilicum var. thyrsiflora* against *St. aureus*, and O*. basilicum var. purpureum* essential oil against *B. subtilis*. The MIC of *O. basilicum var. thyrsiflora* essential oil against *B. subtilis* and MIC of *O. basilicum var. purpureum* against *St. aureus* were twice as high, 6.25 μL/mL. The ampicillin-resistant *E. coli* bacteria also displayed sensitivity against the essential oils tested: thus the MIC values of *O.* x *citriodorum* and *O. basilicum var. purpureum* against those bacteria were 6.25 μL/mL, while *O. basilicum var. thyrsiflora* displayed MIC of 12.5 μL/mL. The action of the essential oils on the all bacteria in this study was evaluated as bactericidal.

All three essential oils have also displayed high antifungal activity, with *O.* x *citriodorum* being the strongest antifungal amongst them: MIC of *O.* x *citriodorum* against *D. hansenii* and *C. guillermondii* were 1.56 and 3.125 μL/mL, respectively (see Fig. 1).

### Radical scavenging activity

**Fig. 2:**
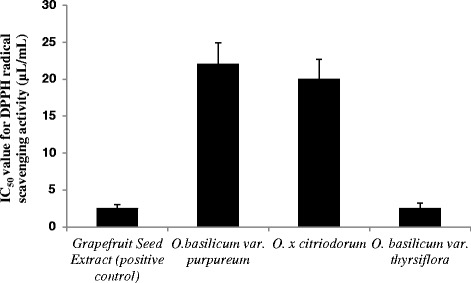
IC_50_ values of antiradical activity of *O. citriodorum*, *O. basilicum* var. *thyrsiflora* and *O. basilicum* var. *purpureum* essential oils. For details, see Methods

The results of radical DPPH assay for of *O.* x *citriodorum*, *O. basilicum var.purpureum, O. basilicum var. thyrsiflora* essential oils are shown on Fig. 2. The highest antioxidant activity was demonstrated by *O. basilicum var. thyrsiflora* essential oil: IC_50_ value for it was equal to the standardized Grapefruit Seed Extract which was used as a control sample (2.5 μL/mL). The antiradical activity for the other two basil species was lower: IC_50_ value for *O.* x *citriodorum* essential oil was 20 μL/mL and for *O. basilicum var. purpureum* was 22 μL/mL. These results were somewhat unexpected, since usually the oils with higher phenolic content are the ones exhibiting higher radical scavenging abilities, whereas in our case the highest antioxidant properties were displayed by the cultivar with the highest linalool (terpene alcohol) content.

### Tyrosinase inhibition activity

**Fig. 3:**
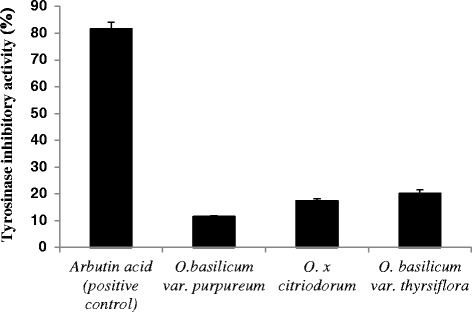
The tyrosinase inhibitory activity of *O. basilicum var. thyrsiflora* and *O. basilicum var. purpureum*, *O. citriodorum* essential oils. For details, see Methods

The enzyme tyrosinase inhibition abilities of all three oils were also assessed as a part of our efforts to find a natural treatment for hyper-pigmentation skin disorder. The values for tyrosinase inhibitory activity of *O. basilicum var. thyrsiflora*, *O. basilicum var. purpureum* and *O.* x *citriodorum* essential oils and arbutin acid (positive control) were calculated to be 20.1 ± 1.4%; 11.5 ± 0.3%; 17.4 ± 0.9% and 81.5 ± 2.6%, respectively (Fig. 3).

## Discussion

Under the experimental conditions of the present study it was revealed that the dominant constituent for *O. basilicum var. purpureum* is methyl chavicol (estragol), whereas the major component for the other variety of the same species, *O. basilicum var. thyrsiflora* is linalool. At the same time, the chemical composition of *O. citriodorium* hybrid plant differed substantially from the first two basil varieties: it had significant aldehyde content, represented by citral, with another prevalent constituent being nerol (monoterpene alcohol). Neither citral nor nerol was detected in the two other species of *Ocimum* (see Table 1). We observed that the essential oil from *O. citriodorum* species displayed the highest antimicrobial activity against the most of microorganisms tested. The experiments showed that essential oils from all three varieties of basil can significantly inhibit the growth of ampicillin-resistant strain of *E. coli* bacteria. It is interesting to notice, that the observed antibacterial activities of the essential oils from O*. citriodorum* and *O. basilicum var. purpureum* against *E. coli* where much higher in case of ampicillin-resistant strain than in the case of a non-resistant one. At the same time, the essential oil from *O. thyrsiflora* cultivar displayed the same, relatively high antibacterial activity in both cases (see Fig. 1).

The essential oils from all three basil cultivars tested showed high inhibition activities against fungi and high radical scavenging activity. Among the three, the essential oil from the *O. thyrsiflora* variety displayed the highest ability to neutralize free radicals and showed results similar to the positive control.

The essential oils from all three varieties exhibited some tyrosinase inhibitory activity, although it wasn’t particularly high.

The essential oils from both *O. citriodorum* and *O. thyrsiflora* varieties of basil show high inhibition rates against *S. aureus* bacteria, which makes it possible to consider using these oils as active natural ingredients for the treatment of skin irritations, since *S. aureus* is extremeley common on the skin of patients with certain dermatological diseases [20], and it is often considered to be a major culprit in causing skin irritation and soft tissue infections [21]. At the same time, the combination of very strong antioxidant properties with some tyrosinase inhibition abilities makes the essential oil of *O. thyrsiflora* a good candidate to be used as a multifunctional cosmetic active in various cosmetic formulas, namely as an antioxidant with some additional skin brightening properties.

## Conclusions

The qualitative and quantitative composition of the three essential oils of three basil cultivars (*O. basilicum var. thyrsiflora*, *O. basilicum var. purpureum* and *O.* x *citriodorum*), cultivated in Armenia, was quite different: *O. basilicum var. purpureum* essential oil contained 57.3% methyl-chavicol (estragol); *O. basilicum var. thyrsiflora* oil had 68.0% linalool, and the main constituents of *O.* x *citriodorum* oil were nerol (23.0%) and citral (20.7%). The presence of thyrosinase inhibitory activity is enhances the pharmacological value of these oils. They had also high antioxidant, antibacterial and antifungal activity and could be used as good sources of natural antimicrobial and antioxidant agents, with possible application in food industry, cosmetics or medicine.

## Abbreviations

DMSO: Dimethyl sulfoxide
DPPH: 1,1-diphenyl-2-picrylhydrazyl
GS: Gas chromatography
MIC: Minimal inhibitory concentration
MS: Mass selective
NIST: National Institute of Standards and Technology
P: Student’s criteria
RRI: Relative retention index
